# Abscisic Acid: Role in Fruit Development and Ripening

**DOI:** 10.3389/fpls.2022.817500

**Published:** 2022-05-10

**Authors:** Kapil Gupta, Shabir H. Wani, Ali Razzaq, Milan Skalicky, Kajal Samantara, Shubhra Gupta, Deepu Pandita, Sonia Goel, Sapna Grewal, Vaclav Hejnak, Aalok Shiv, Ahmed M. El-Sabrout, Hosam O. Elansary, Abdullah Alaklabi, Marian Brestic

**Affiliations:** ^1^Department of Biotechnology, Siddharth University, Kapilvastu, India; ^2^Mountain Research Centre for Field Crops, Sher-e-Kashmir University of Agricultural Sciences and Technology of Jammu, Khudwani, India; ^3^Centre of Agricultural Biochemistry and Biotechnology, University of Agriculture, Faisalabad, Pakistan; ^4^Department of Botany and Plant Physiology, Faculty of Agrobiology, Food, and Natural Resources, Czech University of Life Sciences Prague, Prague, Czechia; ^5^Department of Genetics and Plant Breeding, Centurion University of Technology and Management, Paralakhemundi, India; ^6^Department of Biotechnology, Deen Dayal Upadhyaya Gorakhpur University, Gorakhpur, India; ^7^Government Department of School Education, Jammu, India; ^8^Faculty of Agricultural Sciences, SGT University, Haryana, India; ^9^Bio and Nanotechnology Department, Guru Jambheshwar University of Science and Technology, Hisar, Haryana; ^10^Division of Crop Improvement, ICAR-Indian Institute of Sugarcane Research, Lucknow, India; ^11^Department of Applied Entomology and Zoology, Faculty of Agriculture (EL-Shatby), Alexandria University, Alexandria, Egypt; ^12^Plant Production Department, College of Food and Agricultural Sciences, King Saud University, Riyadh, Saudi Arabia; ^13^Floriculture, Ornamental Horticulture, and Garden Design Department, Faculty of Agriculture (El-Shatby), Alexandria University, Alexandria, Egypt; ^14^Department of Biology, Faculty of Science, University of Bisha, Bisha, Saudi Arabia; ^15^Institut of Plant and Environmental Sciences, Slovak University of Agriculture, Nitra, Slovakia

**Keywords:** abscisic acid, biosynthesis, transport, fruit ripening, development, ethylene, regulatory pathways

## Abstract

Abscisic acid (ABA) is a plant growth regulator known for its functions, especially in seed maturation, seed dormancy, adaptive responses to biotic and abiotic stresses, and leaf and bud abscission. ABA activity is governed by multiple regulatory pathways that control ABA biosynthesis, signal transduction, and transport. The transport of the ABA signaling molecule occurs from the shoot (site of synthesis) to the fruit (site of action), where ABA receptors decode information as fruit maturation begins and is significantly promoted. The maximum amount of ABA is exported by the phloem from developing fruits during seed formation and initiation of fruit expansion. In the later stages of fruit ripening, ABA export from the phloem decreases significantly, leading to an accumulation of ABA in ripening fruit. Fruit growth, ripening, and senescence are under the control of ABA, and the mechanisms governing these processes are still unfolding. During the fruit ripening phase, interactions between ABA and ethylene are found in both climacteric and non-climacteric fruits. It is clear that ABA regulates ethylene biosynthesis and signaling during fruit ripening, but the molecular mechanism controlling the interaction between ABA and ethylene has not yet been discovered. The effects of ABA and ethylene on fruit ripening are synergistic, and the interaction of ABA with other plant hormones is an essential determinant of fruit growth and ripening. Reaction and biosynthetic mechanisms, signal transduction, and recognition of ABA receptors in fruits need to be elucidated by a more thorough study to understand the role of ABA in fruit ripening. Genetic modifications of ABA signaling can be used in commercial applications to increase fruit yield and quality. This review discusses the mechanism of ABA biosynthesis, its translocation, and signaling pathways, as well as the recent findings on ABA function in fruit development and ripening.

## Introduction

Plant physiology has contributed significantly to crop improvement. Knowledge of plant physiology concerning the role and mechanism of plant hormones or growth regulators is significant for crop tolerance to abiotic stresses and resistance to biotic stresses (Neelapu et al., 2021; Pandita and Wani, 2021). Abscisic acid (ABA) is a major phytohormone that plays a crucial role in regulating plant growth and development, stress responses, and multiple physiological processes (Hirayama and Shinozaki, 2007; Yoshida et al., 2019). It is one of the most studied plant hormones for its numerous functions in crops. The role of ABA in combating various abiotic stresses, such as drought (Wright, 1969; Kannangara et al., 1983; Wang et al., 2008), salinity (Khadri et al., 2006; Etehadnia et al., 2008), flood (Fukao et al., 2011; Yeung et al., 2018), heat (Li et al., 2021), and cold (Lalk and Dörffling, 1985; Mohapatra et al., 1988), has been very well established in several crops. The involvement of ABA has been demonstrated not only against abiotic stresses but also its role against biotic stresses as elucidated by several studies (Mohr and Cahill, 2003; Asselbergh et al., 2008). Beyond its function under biotic and abiotic stresses, ABA is controlled by many physiological processes, with ABA playing an important role in fruit development and ripening. It is well known that fruit is a significant source of vitamins and minerals and that it plays an essential role in human nutrition (DellaPenna and Pogson, 2006). Therefore, detailed knowledge of the physiology of fruit is critical to ensure food and nutritional security, given the increasing population of the globe and the limited amount of resources. Fruit development is a unique characteristic of naked-seeded plants where it serves to protect the seeds and aids in their dispersal. Generally, the ovary transforms into a fruit after double fertilization, but in some plants, such as the apple or strawberry, the accessory tissue also transforms into a fruit (Obroucheva, 2014). The development and ripening of fruit follow several different patterns, with ripening involving modification of the cell wall, softening of the fruit, synthesis of pigments, conversion of starch to simple sugars, and synthesis of volatiles that enhance the flavor and aroma of the fruit (Saladié et al., 2007). While the role of ABA has been well established in various biotic and abiotic stresses and several physiological functions, scientists have begun to elucidate its function in fruit development and ripening. Although many previous studies have attempted to explain the function of ABA in the developmental and fruit ripening phases, the results of these studies and their conclusions with a view to the future have not been well-reviewed. Therefore, this article comprehensively reviewed all aspects of ABA, with special emphasis on the process of its biosynthesis, signal transduction, transcriptional regulation, and transport mechanisms. This review manuscript also discusses the role of ABA in seed and fruit development, ripening, bud dormancy, and how it mediates ethylene synthesis and its interactions with other hormones.

## Biosynthesis and Catabolism of Abscisic Acid in Plants

**FIGURE 1:**
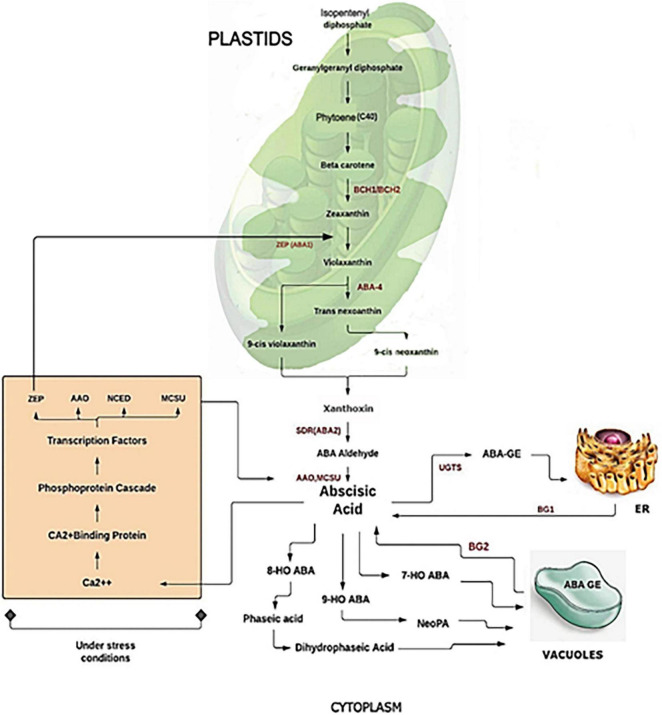
Biosynthesis and catabolism of abscisic acid (ABA).

Abscisic acid refers to a class of metabolites referred to as isoprenoids, also known as terpenoids, whose precursor is isopentenyl (IDP) at the base of five carbons (C5). The synthesis of ABA proceeds from the cleavage of the C40 carotenoid precursor. In this process, the plastid-localized 9-*cis*-epoxycarotenoid dioxygenase (NCED) is the first to be involved, assisting in the cleavage of the epoxycarotenoid precursor to form xanthoxin (Figure 1). Xanthoxin further produces ABA through reactions involving two cytosolic enzymes *via* Abscisic aldehyde (Endo et al., 2014). The primary catabolic pathway in the cytoplasm is the formation of 8′-hydroxy ABA and phaseic acid, which is catalyzed by the cytochrome P450 enzyme ABA 8′-hydroxylase. In addition, there are other alternative catabolic pathways *via* conjugation (Vilaró et al., 2006), 4′-reduction, and 7′-hydroxylation. An alternative pathway for IDP synthesis has also been observed in some Eubacteria (Lichtenthaler, 1999) and fungi (Inomata et al., 2004).

Plastidic isoprenoids along with carotenoids were synthesized from IDP from an MVA-independent pathway, called the 2-C-methyl-d-erythritol-4-phosphate (MEP) pathway (Nambara and Marion-Poll, 2003). Several thorough studies have been conducted on the *de novo* ABA synthesis pathway in various plant species, such as *Arabidopsis*, maize, and tomato, by developing biosynthetic mutants in other organisms as well (Seo and Koshiba, 2002; Sussmilch et al., 2017). Another pathway of free ABA *via* hydrolysis of glucose-conjugated ABA (ABA-GE) to ABA by 2 β-glucosidases was also identified (Dong and Hwang, 2014). The rate of biosynthesis and catabolism of the hormone helps to determine the level of ABA in particular plant tissue. Therefore, it is necessary to understand the genes involved in ABA metabolism to understand its functions. Scientists identified specific metabolic pathways and genes that revealed differential regulation of metabolic pathways that can help to mitigate ABA levels at transcriptional and post-transcriptional levels (Nambara and Kuchitsu, 2011). This balance can occur at multiple levels and considers the expression of the genes involved.

Moreover, recent research has provided new insights into ABA’s regulation and metabolism concerning its physiological roles. Among the various ABA catabolic pathways, recent genomic advances have shown that *Arabidopsis CYP707A* genes encode 8′-hydroxylases of ABA that are responsible for catalyzing the previous step in the ABA catabolic pathway (Nambara and Marion-Poll, 2005). It was determined that ABA catabolism and biosynthesis are closely linked through feedback and feedback loops that limit ABA content, as seen in the fruit ripening process where ABA increases rapidly during early fruit ripening (Liao et al., 2018). Various other studies have revealed a mechanism of ABA biosynthesis and catabolism targeting other metabolic channels. Several inhibitors have been studied that inhibit ABA catabolism in several steps by targeting ABA 8′-hydroxylase, leading to the regulation of ABA content that ultimately promotes plant resistance to drought stress (Kitahata et al., 2005). In *Arabidopsis*, another plant growth retardant, uniconazole, has been identified as a potent ABA inhibitor that has been shown to strongly inhibit 8′-hydroxylase ABA, leading to an increase in plant tolerance to drought (Saito et al., 2006). Further investigation on *Arabidopsis* demonstrated that nordihydroguaiaretic acid (NDGA) serves as an inhibitor of the ABA anabolic enzyme 9-*cis*-epoxycarotenoid dioxygenase (NCED) significantly arrested rice seed germination (Zhu et al., 2009). Diniconazole, a fungicide, acts as a competitive inhibitor of recombinant *Arabidopsis* ABA 8′-hydroxylase, *CYP707A3* (Mizutani and Todoroki, 2006). Due to any injury, the ABA catabolic activity was found to be increased in potato. The increased level of ABA plays a major role in different conditions such as it provides stress tolerance under drought in *Arabidopsis* (Kuromori et al., 2018), stress tolerance at the reproductive stage in cereals (Ji et al., 2011). It also has a role in fruit maturation (Yang and Feng, 2015) such as in mango (Kondo et al., 2004) and woodland strawberry (Liao et al., 2018) and in regulating the fruit quality.

## Abscisic Acid Signal Transduction and Its Transcriptional Regulation

Abscisic acid regulates and controls several critical biological mechanisms in plants through its biosynthesis, accumulation, catabolism, perception, and transmission of crucial information by triggering signaling pathways (Sugimoto et al., 2014; Hou et al., 2020). It plays a central role in adaptive responses to stress, development, growth, and maturation of fruits through signal transduction. ABA biosynthesis and signaling pathways have been significantly characterized in higher plants. Over the past two decades, considerable progress has been made in understanding the mechanism of ABA signal transduction and its transcriptional regulation in fruits. Different target genes, transcription factors (TFs), protein phosphorylases, protein kinases, cis-elements, and ABA signal receptors, especially the essential signal transduction factors, have been discovered in various studies (Cutler et al., 2010; Mou et al., 2016; Lü et al., 2018).

Abscisic acid synthesis is controlled by stimuli from the external and internal environment, and its level is controlled through a dynamic balance between catabolism and biosynthesis. Genetic and biochemical evidence revealed that ABA is synthesized from xanthoxin by oxidative cleavage of ß-carotene by 3-hydroxylase (Iuchi et al., 2001; Ampomah-Dwamena et al., 2009). ABA-aldehyde is formed as an intermediate compound, while 9-cis-epoxycarotenoid dioxygenase (NCED) is an essential enzyme associated in ABA biosynthesis with NCED genes (Wheeler et al., 2009; Zifkin et al., 2012). The NCED enzyme takes 9-cis neoxanthin and 9-cis violaxanthin as substrates, splitting into ABA precursor xanthoxin (Cutler et al., 2010). Any changes in ABA levels lead to upregulation and downregulation of NCED genes through transcriptional control (Yang and Feng, 2015). For example, the *SlNCED* gene mutation in tomatoes reduces ABA accumulation, leading to delayed fruit ripening (Ji et al., 2014). Virus-induced gene silencing of the *FaNCED1* gene in strawberries lowers the ABA level to 46% and produces colorless fruit (Jia et al., 2011). Expression of *FaNCED1* and *FaABA2* genes was increased during strawberry ripening, whereas expression of *FaCYP707A1* and *FaUGT75C1* genes was higher in the absence or at low ABA levels during fruit development. Also, ripening-related TFs such as *FaASR*, *FaMYB1*, *FaMYB5*, and *FaMYB10* genes, but only *FaMYB10* was upregulated during strawberry fruit ripening (Kim et al., 2019). Similar types have been observed in blueberry fruit (Karppinen et al., 2018) and sweet cherry (Shen et al., 2014). Fruit ripening has been influenced by the *PaNCED1* and *PaNCED3* genes through ABA accumulation in avocados (Chernys and Zeevaart, 2000). Recently, the role of NCED genes, including *PpNCED1* and *PpNCED5* were observed in regulating the ABA response during peach fruit ripening. Both genes were upregulated at the start of the ripening process, *PpNCED1* showed a constantly higher expression level than *PpNCED5*, while during post-harvest both these genes showed the same expression patterns. The results revealed that the dynamic response of ABA was cooperatively controlled by these two genes during fruit ripening (Wang et al., 2021).

Abscisic acid signal transduction mechanism is very complex and studied mainly in higher plants to fully understand the fruit development and ripening process. Based on differential studies, it is divided into three main components, namely the ABA receptor proteins Pyrabactin Resistance (PYR)/Pyrabactin Resistance Like (PYL)/Regulatory Component of ABA Receptor (RCAR) (PYR/PYL/RCAR), negative regulators of group A type 2C protein phosphatases (PP2Cs), and positive regulators of subclass III SNF1-related protein kinases 2 (SnRK2s) (Ma et al., 2009; Melcher et al., 2009; Park et al., 2009). Many other ABA receptors have been described for the signal transduction process in plants, but PYR/PYL/RCAR remains the most reliable family of ABA receptors, and various groups have fully characterized its mechanism (Ma et al., 2009; Melcher et al., 2009; Nishimura et al., 2009; Park et al., 2009). Many structural and biochemical analyses of ABA sensing have presented deep insight knowledge about the signal transduction mechanism (Melcher et al., 2009; Santiago et al., 2009) and discovered 14 PYLs (Ma et al., 2009; Park et al., 2009), 10 SnRK2s (Miyazono et al., 2009), and 9 PP2Cs components in *Arabidopsis thaliana* (Nishimura et al., 2009; Umezawa et al., 2009). Besides, several other genes related to the PYR/PYL/RACR family have been discovered in many plants, including grapevine (Boneh et al., 2012), tomato (González-Guzmán et al., 2014), peach (Zeng et al., 2020), citrus (Romero et al., 2012), and strawberry (Hou et al., 2020).

The ABA signaling cascade is started with the sensing of ABA by the PYR/PYL/RCAR receptors (Ma et al., 2009; Park et al., 2009), which resulted in the activation of ion channels and phosphorylation of downstream proteins (Weiner et al., 2010). The PYLs, which are included in the superfamily of START protein, can sense and attach to ABA *via* ligand-binding pockets (Melcher et al., 2009; Nishimura et al., 2009). After binding, PYL can be able to cease the entrance points of ligand-binding pocket which capture the highly conserved β-loops, also termed as “cap” and “lock,” and establish a “cap-lock” interface. This specialized unit connects with PP2C, hinders PP2C involvement, and then releases SnRK2s from dephosphorylation of PP2C (Yoshida et al., 2006; Fujii et al., 2009; Melcher et al., 2009). This activates SnRK2, which then phosphorylates downstream basic TFs with a leucine zipper, including ABA response element-binding factors (AREB/ABF), V-myb myeloblastosis viral oncogene homolog (MYB), ethylene response factor (ERF), and proteins that activate ABA-induced maturation gene expression (Furihata et al., 2006; Klingler et al., 2010). On the other hand, PYLs can also impede the activity of PP2C in the absence of ABA (Hao et al., 2011; Tischer et al., 2017). Additionally, the magnesium chelatase H subunit (CHLH) pathway is another ABA signal transduction mechanism. CHLH senses the ABA signaling and works as antagonistic to WRKY transcription repressors and activates ABA-dependent genes (Shen et al., 2006; Wu et al., 2009).

The above basic signal transduction components have been recognized in many plant species engaged in ABA transcriptional regulation for fruit development and maturation. Among several ABA-dependent receptors (PYR/PYL/RCAR) in climacteric tomato, *SlPYL1-13* interacted with *SlPP2Cs*, and its interaction was enhanced by ABA accumulation. In contrast, in ABA-independent receptors, SlPP2Cs were found to interact with *SlPYL3* and *SlPYL12*, which may be improved in the presence or absence of ABA (Chen et al., 2016). A downstream TF *SlAREB1* has been analyzed to elucidate its role in regulating the tomato ripening genes. Transient overexpression of *SlAREB1* showed an increased level of ABA signals, activating ethylene biosynthesis genes during fruit ripening (Mou et al., 2016). Kai and coworkers analyzed the role of ABA receptor *SlPYL9* in tomatoes which acts as a positive regulator of ABA signal transduction and fruit ripening. Overexpression of *SlPYL9* affects the expression profiles of ABA-responsive genes such as (*SlPP2C1/2/9*, *SlSnRK2.8*, *SlABF2*) and induces different phenotypes. However, fruit ripening was delayed in *SlPYL9* mutant lines, which indicated the prominent role of *SlPYL9* in tomato fruit ripening (Kai et al., 2019).

Zhu et al. (2017) studied ABA signal transduction transcriptional regulation and characterized 17 fruit development and ripening genes, including 6 *MnSnRK2*, 6 *MnPP2C*, and 5 *MnPYL* genes mulberry (*Morus alba* L.). The results indicated the higher transcripts level of these genes during the fruit development and ripening (Zhu et al., 2017). During the ripening of sweet orange, ABA accumulation and *CsPYL4* and *CsPYL5* genes showed opposite expression patterns, while gene expression of *CsSnRK2* continuously decreased in the ripening process (Romero et al., 2012).

The ABA-responsive genes like 6 *PaSnRK2s*, 6 *PaPP2Cs*, and 3 *PaPYLs* genes were identified in sweet cherry, which has a function in transcriptional regulation of ABA in fruit development and ripening (Wang et al., 2015). Shen et al. (2017) reported the ABA-mediated anthocyanins biosynthesis in sweet cherry through the interaction between 6 *PacSnRK2s* with *PacPP2C1* (Shen et al., 2017). Similarly, the expression of ABA signaling and metabolic genes were investigated during fruit development. The results revealed that the expression level of *PaPP2C1/2/3* were increased in young fruit and ovary while *PaPP2C3/4* were highly expressed in the stamen. On the other hand, 6 *PaSnRK2s* showed varied expression profiles such as *PaSnRK2.1/2.2/2.4* were expressed greatly in young fruit and ovary and *PaSnRK2.1/2.3* in the stamen (Leng et al., 2018). PaSnRK2.1/2.2/2.4 were highly expressed in the ovary and young fruit, while PaSnRK2.1/2.3 were highly expressed in the stamen.

Wang and colleagues explored the interaction between ethylene response transcription factor *PpERF3* and ABA biosynthesis genes *PpNCED2/3* in peach to study the fruit ripening process (Wang et al., 2019). In another study, Zeng et al. (2020) demonstrated the role of ABA in characterizing the signal transduction pathway and transcriptional regulation of 7 *PpSnRK2*, 10 *PpPP2C*, and 7 *PpPYR* during peach fruit development and ripening. The expression profiles of *PpPYR2*, *PpPYR6*, and *PpPYR7* reduced in higher accumulation of ABA during ripening, while transcript expression of *PpPP2C4*, *PpPP2C9*, *PpPP2C10*, *PpSnRK2.4*, and *PpSnRK2.6* were upregulated in response to increased ABA levels (Zeng et al., 2020).

Li et al. (2019) proposed non-climacteric fruit ripening in strawberries and discovered the ABA-FaPYR1-FaPP2C-FaSnRK2 signal transduction system (Li et al., 2019). Two groups studied the strawberry fruit ripening and found that a leu-rich repeat receptor-like protein kinase (*FaRIPK1*) and a sigma factor (*FaSigE*) are involved in ABA transcriptional regulation and interact with *FaABAR* to promote the fruit ripening (Zhang et al., 2017; Hou et al., 2018b). The overexpression of the *FvTCP9* TF gene promoted the fruit ripening in strawberries and function in the ABA regulation and biosynthesis (Xie et al., 2020). Detailed analysis of ABA networks involved in strawberry ripening has unveiled that FaABI1 negatively regulates and *FaPYR1* positively regulates, and ABA-FaPYR1-FaABI1-FaSnRK2 is the core mechanism controlling this mechanism (Chai et al., 2011; Jia et al., 2011, 2013; Li et al., 2011; Han et al., 2015). Furthermore, TF FaMYB10 triggers the ABA signal transduction mechanism to synthesize anthocyanin during ripening (Medina-Puche et al., 2014). Recently, the mechanism of ABA signal transduction during fruit ripening has been investigated in strawberries. The results showed the upregulation of *FaABI1/FaPP2C16/51/16L2/16L1/37* and *FaPYL2/4/8/9/11/12* at the transcript level. The interaction between *FaABI1* and *FaPYL2* might be crucial in fruit ripening (Hou et al., 2020).

## Transport of Abscisic Acid

**FIGURE 2:**
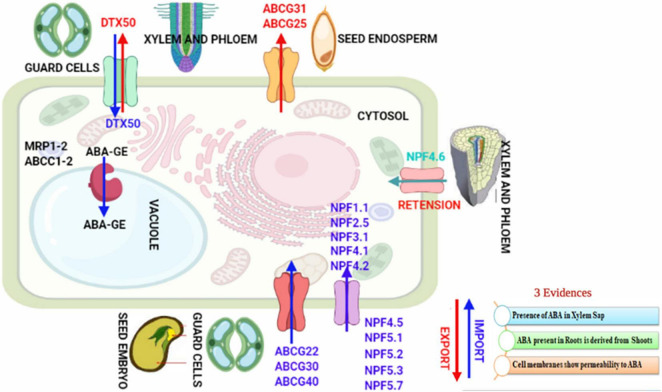
Transporter proteins of ABA.

Abscisic acid has multiple sites of biosynthesis (roots and shoots or leaves) and transporters. Therefore, regulation of the transport of ABA forms complex networks (Kuromori et al., 2018). Inside the fruits (site of action), transport of ABA signaling molecules mainly takes place from the shoot (site of synthesis) (Leng et al., 2013). Inside the fruits, ABA receptors decode the message (distinct from that in other organs of root, stem, or leaf) during the inception of fruit maturation and promote it significantly (Sauter et al., 2001). Phloem exports maximum quantity of ABA during stone formation and initial stages of fruit expansion of developing fruit. Phloem ABA export decreases prominently in later stages of fruit maturation, leading to ABA accumulation in ripening fruit (Jia et al., 2011). Ripening of fruit is synchronized with ABA, and ABA levels within fruit increase significantly in the final phase of maturity (Ren et al., 2011). In *Prunus avium* L. fruit, ABA is transported only *via* the vascular phloem (Else et al., 2004). ABA is freely imported from the shoot into the fruits during the beginning of ripening (Li et al., 2013). From the site of synthesis, ABA transports to the ABA site of action. The auxin transport from shoot apex to root apex and switching at root tips are critical for plants’ involuntary and plastic polarity. The transport of ABA has been studied comprehensively in plants (Sauter et al., 2001; Wilkinson and Davies, 2002; Jiang and Hartung, 2008), but still regulation of ABA transport and its significance in monitoring physiological responses is vague. The three pieces of evidence that confirm the plasma membrane crossways transport of ABA are shown in Figure 2.

The xylem vessels include dead cells and lack the potential to biosynthesize the ABA. The presence of ABA in the xylem sap, therefore, signposts that ABA has been transported from different plant cells. A significant fraction of ABA present in roots is consequential of shoot biosynthesis. The plasma membranes of the plant cells possess ABA permeability (Ikegami et al., 2009; Ernst et al., 2010; Goodger and Schachtman, 2010). The transfer of ABA synthesized in roots has been considered in the context of root-to-shoot signaling or root-derived signal(s), which involves the transfer of ABA through root xylem vessels to shoots, inducing stomatal closure in plant leaves under drought stress (Schachtman and Goodger, 2008) e.g., in Malus × domestica, *Helianthus annuus* and *Acer pseudoplatanus* (Wilkinson and Davies, 2002). However, the stomata close even in deficiency of root-derived ABA (Holbrook et al., 2002; Christmann et al., 2007) and can be initiated only by ABA biosynthesized in the leaf guard cells (Tan et al., 2003; Koiwai et al., 2004; Christmann et al., 2005). ABA concentrations remain more in leaves than roots during drought. ABA buildup in roots occasionally depends on basipetal transport of ABA from aerial shoots (Manzi et al., 2015, 2016). Studies on the ABA deficit mutants and wild species of tomato and *Arabidopsis* concluded that the genotype of shoot influenced the closure of stomata, but not the genotype of root (Holbrook et al., 2002; Christmann et al., 2007). ABA levels improved in leaves (shoots) and roots of *Arabidopsis* seedlings under drought because ABA biosynthesized in the aerial leaves gets transported to the basal roots. However, when shoots and roots were disconnected and subjected to water deficiency independently, ABA amassed mostly in shoots (Ikegami et al., 2009). There are two models for the trans-membrane transport of ABA. These are firstly through the process of passive diffusion and secondly with the help of transporters. ABA must cross the cell lipid membrane passively by diffusion, or the uptake of ABA is mediated by ABA transporters (Jiang and Hartung, 2008).

### Passive DIffusion-mediated Abscisic Acid Transport

Biophysical including physiological investigations of ABA in plant roots and leaves resulted in “ionic trap model.” The ionic trap model elucidates ABA flux between plant organs (Slovik et al., 1995; Wilkinson and Davies, 1997; Sauter et al., 2001; Dodd et al., 2003; Jia and Davies, 2007; Wilkinson et al., 2007). The biophysical aspects suggest that ABA, which is a weak acid, remains in equilibrium at pH 4.7 (pKa: 4.7) between anionic deprotonated (ABA– is non-diffusible form) form and protonated (ABA-H is diffusible form) state with the same concentration (50% of ABA– form and 50% of ABA-H form). Uncharged protonated ABA diffuses readily through the lipid bilayer membrane. The transport of ABA was probed comprehensively in the 1970s and across membranes believed to be due to diffusion. As regards ABA (pKa: 4.7), the cell pH (7.5) and apoplastic pH (5 to 6.1) indicates that most of ABA pools are in ABA–, charged and non-diffusible form due to constant diffusion of ABA-H form (i.e., it acts as a trap) from cytosol through membranes. Consequently, diffusion through membrane lipid bilayer is a limiting step for ABA transport (Kaiser and Hartung, 1981). Xylem sap progressively depletes ABA by diffusion to vascular parenchyma cells, mesophyll, and bundle sheath cells, and nearly no ABA remains in the sap of xylem tissue after it reaches into leaf guard cells (Wilkinson and Davies, 1997; Dodd et al., 2003).

### Transporter-mediated Abscisic Acid Transport

Protein-mediated ABA transport by saturable components (transporters) was first demonstrated in 1980 in specific zones of the plant root, and their presence in mediating cellular uptake of ABA was experimentally characterized (Astle and Rubery, 1980, 1983; Baier et al., 1990; Bianco-Colomas et al., 1991; Windsor et al., 1992; Perras et al., 1994). Localized transporters import ABA into cells better, which accesses intracellular receptors in limited diffusion (Iyer-Pascuzzi et al., 2011). Diverse families of auxin transporters were identified in 1996, and the first breakthroughs in molecular cloning of plasma membrane-localized ATP BINDING CASSETTE (ABC) G25 and G40 (efflux ABA transporter *AtABCG25* and high-affinity influx (+)-ABA transporter *AtABCG40*) plasma membrane-localized ABA transporters of *Arabidopsis* accomplished in 2010 (Verrier et al., 2008; Kang et al., 2010; Kuromori et al., 2010). Plant ABA transporters include ATP binding cassette (ABC) family, nitrate transporter 1 family (NRT1) or nitrate and peptide transporter family (NPF), and detoxification efflux carrier proteins (DTX) (Figure 2; Léran et al., 2014; Chiba et al., 2015; Corratgé-Faillie and Lacombe, 2017; Fan et al., 2017). Biochemical analysis shows that AtABCG25 belonging to the ABCG family, which encodes an ABC transporter (Kuromori et al., 2010) is an exporter and exports ABA from vessels (vascular tissues) to outside of the cell, whereas AtABCG40, also belonging to the ABCG family, is an Importer uptakes ABA from the outside to the inside of the cells (broad, highest in guard cells) (Kang et al., 2010). The abcg40-knockout Arabidopsis mutants were less sensitive to ABA for seed germination and lateral root development and less tolerant to drought (Dreyer et al., 1999). Seed germination of AtABCG25 (*At1g71960*) and AtABCG40 (*At1g15520*) mutant plants (Kang et al., 2010; Kuromori et al., 2010), showed changes in sensitivity to exogenous ABA. The *AtABCG25* and *AtABCG40* loss-of-function mutants showed phenotypes different from the ABA-deficient mutants, suggesting a passive mechanism of ABA transport or that ABA transport *via* transporters is highly redundant or that plasma membrane-localized ABA receptors may confer physiological responses in cells (Pandey et al., 2009). Nitrate transporter NRT1.2 ABA-IMPORTING TRANSPORTER 1 (AIT1), a member of the NRT1/PTR family localized in the plasma membrane, is both the ABA transporter with a higher influx transport activity for (+)-ABA than for (–) - ABA and nitrate (NO3 –) transporter (Huang et al., 1999; Tsay et al., 2007; Kanno et al., 2012). NRT1.2 transfers ABA from xylem vessels to other inflorescence tissues (Kang et al., 2010; Kuromori et al., 2010; Kanno et al., 2012). The nrt1.2 mutants were more ABA sensitive, inhibited germination of seeds, and had increased stomatal aperture on stems resulting in lower inflorescence stem temperature (Kanno et al., 2012). Exogenous ABA-mediated expression of NRT1.2 permitted the entrance of ABA. ABA then promoted binding of PYR–ABA receptor (PYR1) to P-type 2C protein phosphatase (PP2C) and restored BETA-GALACTOSIDASE 4 (GAL4) transcription factor. Cytosol localized PYLs/PYR/RCAR conserved family of proteins are soluble proteins that act as ABA receptors (Park et al., 2009). Furthermore, AIT2 (*At1g27040*), AIT3 (*At3g25260*) (does not discriminate between (+) and (–)-ABA), and AIT4 (*At3g25280*). ABA transporters of PTR family members localized at plasma membrane were also identified (Kanno et al., 2012). AtABCG25 and AtABCG31 of ABCG subfamily are localized in the seed endosperm and export ABA from the endosperm, whereas AtABCG30 and AtABCG40 of ABCG subfamily localized in embryo imports ABA into an embryo of the seed, and their coordinated activity suppresses extension of radicle and succeeding embryonic growth (Kang et al., 2015). ABA conjugates shuttle the signal of ABA between organs (Nambara and Marion-Poll, 2005; Jiang and Hartung, 2008). ABA-glucose ester (ABA-GE) requires glucosyl-transferase for synthesis and b-glucosidases for the dissociation to ABA and is the most plentiful conjugated form of ABA. Levels of ABA-GE increase in xylem sap upon water deficiency (Hansen and Dörffling, 1999). ABA-GE transporters have been hypothesized as ABC transporters (Sauter et al., 2002) but have not been established. *AtDTX50* belonging to the MATE gene family localized in plasma membrane shows expression in guard cells and vascular tissues and acts as an exporter. The *AtDTX50* overexpression experienced plants’ rapid wilting under drought stress and knockout phenotypes showed growth arrest and drought tolerance (Zhang et al., 2014).

## Role of Abscisic Acid in Seed Development

There is a complex network of genes responsible for ABA biosynthesis and catabolism, which includes multiple level gene regulation under the influence of environmental changes. Seed is a vital source for conserving germplasm and plant biodiversity (Nambara et al., 2010). Researchers have revealed several factors that take part in ABA synthesis and metabolism and its transcription and signaling (Holdsworth et al., 2008; McCourt and Creelman, 2008). Seed dormancy is a critical stage for plants and an adaptive parameter in various seed crop species that enables crop plants’ survival under stressed conditions. Seed dormancy is classified as primary and secondary seed dormancy that is chiefly regulated *via* ABA and gibberellin (GA), which are again primarily found to control the equilibrium between seed dormancy and germination. Metabolism of ABA should be highly regulated and should not depend only on seeds in case of Seed dormancy. Maternal ABA is responsible for inducing primary seed dormancy in plants. ABA performs a sequence of vital physiology roles in higher plants, out of which one role is to induce bud and seed dormancy, quicken foliage fall, and promote stomatal closure. ABA induces maturation and dormancy by regulating different transcription factors such as LEAFY COTYLEDON1 (LEC1) and LEC2, which basically restricts the germination process of the developing embryo. FUSCA3 and ABSCISIC ACID INSENSITIVE3 (ABI3) are associated with inhibiting premature germination by reserving accumulation (Mönke et al., 2012; Yan and Chen, 2017). The expression of *ABI3* is retained in the seed at a higher level until the final maturation stage arrives (Perruc et al., 2007). Furthermore, other late embryogenesis abundant proteins viz., ABI3 and LEC1 were involved in regulating the expression of genes associated with storage reserve accumulation and acquisition of desiccation tolerance, such as late embryogenesis abundant proteins (Parcy et al., 1994). (Finch-Savage and Leubner-Metzger, 2006) observed that ABA is capable of obstructing germination by water uptake prevention and endosperm rupture. After the restoration of the favorable conditions in seed, the ABA level gets decreased, and gibberellic acid (GA) increases, which further allows the embryos to expand and break dormancy (Manz et al., 2005). Studies in *Arabidopsis* genes *NCED6* and *NCED9* indicated that the synthesis of ABA in the endosperm is chiefly accountable for seed dormancy (Lefebvre et al., 2006). Furthermore, it was found that the seed dormancy is in control of protein Delay of germination 1 (DOG1), which further demands PP2C phosphatases of the ABA signaling pathway (Née et al., 2017). Another protein HONSU was also found in *Arabidopsis*, performing the same function (Kim et al., 2013). Histone demethylases LDL1 and LDL2 and *Arabidopsis* zinc-finger gene, Mediator of ABA-regulated dormancy *1* (*MARD1*), were also studied in *Arabidopsis* to control seed dormancy by regulating regulation DOG1 (He and Gan, 2004; Zhao et al., 2015). Three *Arabidopsis* Protein Kinases *viz.*, SnRK2, SRK2D/SnRK2.2, SRK2E/SnRK2.6/OST1, and SRK2I/SnRK2.3, are associated with ABA signaling were found vital for the regulation of seed development and dormancy (Nakashima et al., 2009).

Apart from the model species ABA also plays a significant role in the seed development of fruits. For example, Amritphale et al. (2005) reported that the envelope formed due to ABA biosynthesis activity reduces the pre-germinative process in radicles and the perisperm-endosperm structure regulates the mature seed development in cucumber. ABA along with sugar controls the germination and seed dormancy after the onset of ripening berry fruit and several gene families have been identified including WRKY transcription factors and PP2C protein phosphatases (Gambetta et al., 2010). Wang and colleagues identified the key genes such as *PpNCEDs* and *PpCYP707As* which have functions in the seed and bud dormancy in peach. In another study, Chen et al. (2008) revealed that the higher concentration level of ABA in the endocarp of the red bayberry promotes the seed dormancy and inhibits the seed germination inside the fruit.

E3 ligase, a chief member in Ubiquitination, plays an important role in regulating the ABA signaling process, de-repression, ABA response activation, and degradation of signaling components. TaPUB1 helps reduce the sensitivity of wheat seedlings toward ABA and eventually serves as a negative regulator in the signaling pathway of ABA during the interaction with TaPYL4 and TaABI5, which finally affects the seed development process of wheat (Zhang et al., 2021). Moreover, it’s even been found that seeds treated with ABA can delay germination in bluebunch wheatgrass (Richardson et al., 2019). Further studies also proved that ethylene and nitric oxide (NO) counteract the function of ABA in seeds and enhance the dormancy release as well as germination process (Arc et al., 2013; Signorelli and Considine, 2018). Seed dormancy was also affected by cold treatment as seen in *Pseudotsuga menziesii* by changing ABA level (Corbineau et al., 2002), increased chilling in western white pine (*Pinus monticola)* was also found helpful in dormancy termination (Feurtado et al., 2004). A seed-specific peroxiredoxin, AtPER1, was found to enhance seed dormancy by eliminating ROS to repress ABA catabolism and GA biosynthesis, and hence improvises the primary seed dormancy and lessens the sensitivity of seeds upon harsh environmental conditions (Chen et al., 2020a). Another gene in Rice *Oryza sativa Viviparous1* (*OsVP1*) activates gene *Seed dormancy 4 (sdr4)* which controls rice seed dormancy again by impacting the ABA signaling pathway (Chen et al., 2021). H_2_O_2_ was found to affect the regulation of ABA catabolism and GA biosynthesis during seed imbibitions, upregulated ABA catabolism genes (e.g., *CYP707A* genes), resulting in a decreased ABA content during imbibitions and thus exerted control over seed dormancy and germination (Liu et al., 2010). Furthermore, it was observed that ABA catabolism is rapid enough to play an important role in the regulation of ABA accumulation as studied in the maize plants (Ren et al., 2007). Again in the case of cereals, investigation in barley coleorhiza proved that the *ABA 8*′-*hydroxylase* gene plays a significant role in seed dormancy release (Kępczyński et al., 2021).

## The Regulatory Roles of Abscisic Acid in Fruit Development and Ripening

**FIGURE 3:**
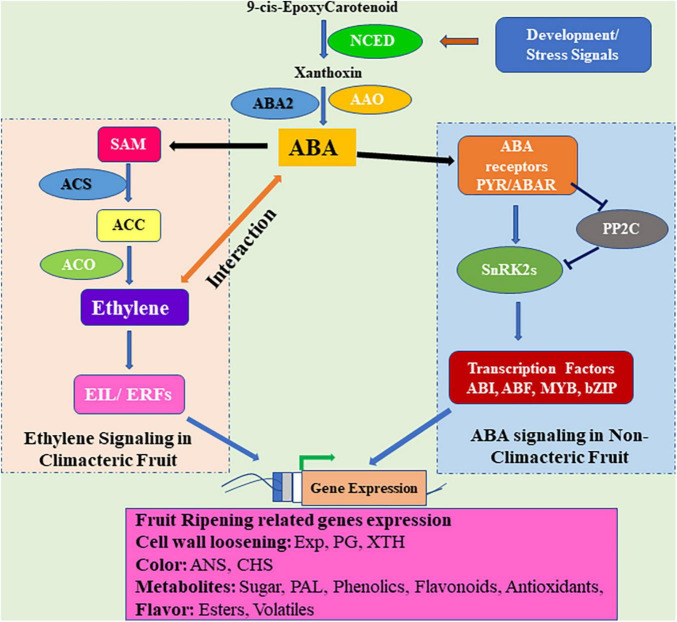
Abscisic acid and ethylene signaling in the regulation of fruit ripening. In climacteric fruits, ABA cross-talks with ethylene and regulates ethylene synthesis. ABA signal is perceived by the PYR1–PP2C–SnRK2 module which regulates the phosphorylation of ABA-responsive transcription factors (TFs) and induces the expression of genes related to development and ripening in non-climacteric fruits. Ethylene synthesis is catalyzed from SAM by ACS and ACO enzymes. Ethylene binding activates EIN3/Ethylene Insensitive3-Like (EIL) transcription factors, which can activate the expression of ethylene response factor (ERF) TFs. ERFs modulate the ethylene-regulated genes by binding to their promoters. ACC: 1-aminocycloproane 1-carboxylic acid, ACS: ACC synthase, ACO: ACC Oxidase, SAM: S-adenosyl methionine, PYR: Pyrabactin Resistance, PP2C: Type 2C protein phosphatases, SnRK2C: SNF1-related protein kinases 2, EXP: Expansins, PG: Polygalacturonase, XTH: Xyloglucan hydroxylase, NCED:, AAO:, ABA2: ANS: Anthocyanin synthase, CHS: Chalcone synthase, ABI: ABA insensitive, ABF: ABA-response element binding factors.

In recent years, research on the role of ABA in plant systems has expanded to fruit growth and development, ripening, and senescence. Fruits are divided into two kinds: climacteric and non-climacteric, based on the maturation process, respiration, and ETH production (Giovannoni, 2001). In the climacteric fruit, ETH is responsible for the start of the ripening process, and maturity (Giovannoni, 2001; Zaharah et al., 2012). Apart from ethylene, the regulatory effect of ABA in the ripening process of climacteric and non-climacteric fruits has been reported to be essential. ABA and ETH are thought to have a synergistic effect, and their interaction leads to control the fruit ripening (Figure 3). Exogenous ABA has been shown to accelerate the maturation of various climacteric fruits (e.g., tomato, banana, peach, mango, and melon) through modulatory biological effects on numerous ripening-related processes in a number of studies (Zhang et al., 2009a,b; Zaharah et al., 2013; Chen et al., 2016; Mou et al., 2016). Exogenous administration of ABA increases the rate of ethylene synthesis and respiratory activity in fruits and speeds up the ripening process (Zhang et al., 2009a). The regulatory role of ABA during fruit ripening has been validated by studies on both climacteric and non-climacteric fruits. The ABA is mainly involved in the ripening of non-climacteric fruits such as strawberries, grapes (Owen et al., 2009; Zhang et al., 2009a), and blueberry (Zifkin et al., 2012), it has also been found to be involved in the ripening of climacteric fruits such as peach (Zhang et al., 2009a), tomato (Galpaz et al., 2008) and banana (Jiang et al., 2000). The accumulation of ABA occurs in climacteric fruits prior to the production and release of ethylene indicates that ABA is an upstream regulator of ethylene biosynthesis and responses (Leng et al., 2009; Wang et al., 2013; Mou et al., 2016). ABA is observed to negatively regulate the synthesis of ethylene until its endogenous level reaches the peak; thereafter, at the start of the ripening process, the ABA positively regulates ethylene biosynthesis (Sun et al., 2010). ABA mediates the transformation of 1-aminocycloproane 1-carboxylic acid (ACC) to ethylene during fruit ripening *via* ethylene-dependent or -independent mechanisms (Jiang et al., 2000; Zhang et al., 2009b; Zaharah et al., 2013).

### Abscisic Acid in Development and Ripening of Climacteric Fruit

The ripening process in climacteric fruits (fleshy fruits like banana, melon, pear, and tomato) requires a high rate of ethylene production followed by a high rate of respiration (Biale, 1964; McMurchie et al., 1972). Whether ABA is the upstream regulatory signal necessary for ETH production was previously unknown. ABA levels peak earlier than ethylene levels during the ripening phase of climacteric fruits, including peach, tomato, and banana, corresponding to ABA’s role in regulating ethylene production. Reduced ABA accumulation and increased shelf life of tomato fruits with improved firmness result from suppressing *NCED* gene expression in tomato fruit (Sun et al., 2012).

The exogenous application of ABA increases its endogenous levels and ethylene production by regulating ETH biosynthesis pathway genes, ACO1, and ACS2 expression, during the maturation of climacteric fruits (Zhang et al., 2009a). Application of ABA biosynthesis inhibitors like Fluridone or NDGA to ripening tomato fruits reduces the expression of ETH biosynthesis genes *ACO1* and *ACS2*, and fruit ripening is delayed (Zhang et al., 2009a; Zaharah et al., 2012; 2013). Overexpression of the *NCED* gene promotes ABA synthesis and enhanced drought tolerance in tomato ripening mutants (Thompson et al., 2007). *NCED* transcript levels are reduced in tomato NCED RNAi plants, and genes involved in cell wall loosening, such as -galactosidase, polygalacturonase, and expansins are downregulated, extending the fruit’s shelf life (Sun et al., 2012). The mechanism of ABA-mediated ethylene production during climacteric fruit ripening has not been well investigated, and more research is needed.

### Abscisic Acid in Development and Ripening of Non-climacteric Fruit

Non-climacteric fruits (grape, citrus, and strawberry) are ripe without ethylene and reduced respiration rate. Although ethylene accumulation is minimal during non-climacteric fruit ripening, ABA levels reach a high; hence, ABA plays an essential role in the ripening and senescence process in non-climacteric fruits (Symons et al., 2006; Cantín et al., 2007; Shalom et al., 2014). ABA metabolism, transport, and signaling determine the amount of ABA in fruit or cell. ABA level was observed higher in immature melon (*Cucumis melo* L.) fruit. It steadily declines with the maturation of the fruit, whereas ACC content and ACC Oxidase (ACO) activity remain low until the ABA level reaches its max (Sun et al., 2013). *NCED*, an ABA biosynthesis gene, was expressed at a higher level in peaches and grapes at the onset of ripening and elevated to the highest till harvesting (Zhang et al., 2009b).

Exogenous ABA treatment enhances fruit ripening and softening in non-climacteric fruits by upregulating the production of ABA, ETH, and cell wall modifying enzymes. However, ABA biosynthesis inhibitors (NDGA and Fluridone) slow down the ripening process. The ABA content of strawberry fruit was reduced by RNA interference (RNAi)-mediated inhibition of *FaNCED1*, resulting in delayed ripening and uncolored fruits, which could only be restored by adding exogenous ABA (Jia et al., 2011). Applying ABA at the turning stage can promote cucumber fruit ripening, while endogenous ABA level reaches highest in pulp before the fruit gets fully ripe (Wang et al., 2013). Proteomics study on grape ripening revealed ABA-induced expression of regulatory proteins involved in fruit ripening network at the start of the ripening stage (Giribaldi et al., 2010).

In strawberry, fruit expansion, chlorophyll degradation, and ripening is regulated mainly by ABA, and ethylene plays a minor role (Ji et al., 2012). The transcript level of NCED2 in strawberry pulp from different development stages was found consistent with the level of ABA content, the crucial role in ABA biosynthesis in strawberry fruit as well as in avocado (Chernys and Zeevaart, 2000), blueberry (Zifkin et al., 2012); hillberry (Karppinen et al., 2013); grape (Zhang et al., 2009a); orange (Rodrigo et al., 2006). RNAi mediated silencing of a putative ABA receptor magnesium chelatase H subunit (FaCHLH/ABAR) in strawberry fruits resulted in a reduction in anthocyanin accumulation and ripening (Jia et al., 2011). The expression of ABA biosynthesis genes *VmZEP*, *VmSDR*, and *VmAO* are the highest before the ripening stage in the berry, whereas *VmNSY* and *VmNCED1* expression is low in the early stages and tends to increase during the ripening stage, indicating that the pulp maintains a higher level of ABA than the fruit skin and seeds (Karppinen et al., 2013).

## Abscisic Acid-Mediated Regulation of Ripening Related Metabolic Pathways

Apart from fruit development, ABA has been essential in stimulating the biosynthesis of anthocyanins, flavonoids, and polyphenols in research involving several metabolic pathways. The carotenoid and xanthophyll biosynthesis routes are located upstream of the ABA biosynthesis pathway; consequently, the carotenoid and ABA biosynthesis processes may act in tandem throughout the ripening process. ABA and its catabolic genes, such as 9-cis-epoxycarotenoid dioxygenase (*ClNCEDs*) and Abscisic Acid 8’-Hydroxylase (*ClCYP707As*), regulate carotenoid production during ripening (Wang et al., 2017). Increased lycopene accumulation in sweet watermelon is positively associated with the expression of the phytoene synthase *ClPSY1* (Wang et al., 2017). The synthesis of ABA in grapes begins with the onset of ripening. An increase in ABA concentration is linked to increased expression of the anthocyanin biosynthetic genes *VmCHS* and *VmANS* (Jaakola et al., 2002). The most significant levels of ABA receptor *VlPYL1* expression were seen in a grape leaf and fruit tissues, which rose throughout fruit development but decreased as the fruit matured. In grape berries, transient expression of *VlPYL1* increased anthocyanin accumulation (Gao et al., 2018).

The role of ABA in the ripening of fruits is also linked to the sugar signaling system. ABA controls metabolic processes such as photosynthate availability and produces phenylpropanoid metabolites during fruit ripening (Jia et al., 2013; Wang et al., 2017). The role of ABA in the unloading of photosynthate from phloem to developing fruits and enhancing the sugar accumulation into apple vacuoles and glucose and fructose content in developing citrus fruit without affecting the organic acid content has been reported (Yamaki and Asakura, 1991). In sweet cherries and peaches, higher levels of ABA correlate with higher levels of ethylene and sugar-to-acid ratio (Kondo and Gemma, 1993; Zhang et al., 2009b). ABA spraying of “Hakuho” peaches at the beginning of ripening increased sugar content by increasing ABA levels (Kobashi et al., 1999). The accumulation of the sugar in fruits can be said to be the interaction of ABA and sugar signaling pathway during the ripening process (Bastías et al., 2011) and release of sugar from storage carbohydrates or import of sugar from distal organs *via* phloem transport.

Fruit color is an essential criterion for purchasing fruits because pigment accumulation is strongly connected to the amount of ABA in the fruit (Jia et al., 2016; Pilati et al., 2017). ABA causes anthocyanin buildup in fruit, which causes coloration, and stimulates fruit defense by producing phenolics, which function as antioxidants (Lacampagne et al., 2010). In strawberries, ABA treatment increased anthocyanin, Phenyl Ammonia Lyase activity, and phenolic compounds, whereas, in grapes, ABA treatment increased flavonoids, anthocyanins, total phenolic content, and antioxidants (Jiang and Joyce, 2003; Cantín et al., 2007; Sandhu et al., 2011). The enzyme UDP-glucose: flavonoid 3-O-glucosyltransferase (UFGT) is involved in anthocyanin biosynthesis, and ABA treatment of grapes increased the expression of UFGT and the production of various anthocyanins such as cyanidin, delphinidin, petunidin, and malvidin during the ripening phase (Jeong et al., 2004). The coloring of ABA-treated grapes was shown to be superior to control or ethephon-treated grapes even after cold storage, suggesting that ABA might be utilized as an alternative to ethephon to improve the quality of “Crimson Seedless” grapes. The buildup of ABA during fruit development in the bilberry (*Vaccinium myrtillus* L.) increased the expression of the *VmANS* and *VmCHS* genes (Karppinen et al., 2013). ABA action upregulates transcriptional regulators such as *ArEB1*, *FaMYB10*, *PacMYBA*, and structural genes involved in the phenylpropanoid and flavonoid pathways, as well as genes involved in acylation and anthocyanin transport into the vacuole, in tomato, strawberry, cherry, and grape (Koyama et al., 2010; Berli et al., 2011; Bastías et al., 2014; Chen et al., 2014).

The use of ABA at the pre-harvest stage enhanced the phenolic content of several grape varieties (Cantín et al., 2007; Sandhu et al., 2011). The effect of ABA in lowering tannin concentration in grape skin has also been explored, as it inhibits the actions of LAR and ANR (Lacampagne et al., 2010). During the ripening of climacteric fruits, aromatic volatiles such as esters are formed, and ethylene is involved in their production (Yang et al., 2016). The use of ABA increased the expression of ester biosynthesis genes such as *MdAAT2* and *MdBCAT1*, resulting in increased production of hexyl propionate and ethyl-2-methyl butyrate in apple (Wang et al., 2018). Exogenous application of ABA in grape berries caused a change in the composition of volatile compounds such as C6 aldehydes and alcohols during ripening (Kalua and Boss, 2009; Ju et al., 2016). Meanwhile, high endogenous ABA levels in grape and strawberry fruits modulated the aromatic pathways for alcohol acyltransferase (AAT) and alcohol dehydrogenase (ADH) (Deluc et al., 2009; Jia et al., 2016).

## Role of Abscisic Acid in Bud Dormancy

Bud dormancy is a complex trait and crucial physiological mechanism in plants, especially perennial fruit species. Bud dormancy assists vegetative bud to cope with the extreme cold conditions and survive to continue the phenology cycle which allows determining the bud growth resumption, flowering, and fruiting in the coming season (Beauvieux et al., 2018). Bud dormancy is classified into three types, namely ecodormancy, endodormancy, and paradormancy. Due to climate changes the endodormancy release may be affected by the less chilling period in coming years which directly influences the flowering and can cause substantial loss in fruit yield (Pan et al., 2021). During bud dormancy, significant molecular and physiological changes occurred such as changes in hormonal levels, restricted import of sucrose, slow cell division process, and deposition of plasmodesmatal callose. Great efforts have been exhausted to study the different aspects of bud dormancy and a diverse range of techniques have been applied to unveil the complex molecular pathways controlling this complex trait (Beauvieux et al., 2018).

Abscisic acid is considered as an important phytohormone regulating and the bud dormancy in plants especially maintaining the endodormancy in fruit species (Zheng et al., 2015). It has been unraveled that ABA as major hormone crosstalk with other endogenous phytohormones and chemicals and integrates with environmental cues to govern the bud dormancy. For example, karrikin and SL have been controlled by two receptors *KARRIKIN INSENSITIVE2* (KAI2) and *DWARF14* (D14) which are homologous and are involved in bud dormancy (Zhao et al., 2013; Wang et al., 2020). However, the crosstalk between these two receptors and ABA during endodormancy still needs to be elucidated. It is reported that increasing levels of ABA and low temperatures promote endodormancy. Yang et al. (2018) argued that a special type of *HD-Zip genes* expressions is influenced by ABA during bud dormancy. Total 47 *HD-Zip* genes were identified in pear which in connection to ABA regulate dormancy transition. On the other hand, the ABA concentration is reduced in buds due to low-temperature exposure for an extended period which results in the break of endodormancy. ABA concentration enhanced during the onset of endodormancy and reduced its level following the bud dormancy break in pear. About 39 candidate genes related to signal transduction and ABA metabolism have been identified and found increased expression of *PpCYP707A-3* but declined ABA contents in pear buds (Li et al., 2018).

Zheng et al. (2015) demonstrated that *VvNCED* and *VvXERICO* transcript levels in ABA metabolism regulate the bud dormancy in grapes. Similarly in another experiment, Zheng and colleagues demonstrated that the overexpression of *VvCYP707A4* reduces ABA concentration and helps to release the bud dormancy in grapes (Zheng et al., 2018). Decreasing the ABA level during the bud dormancy process was correlated with reduced *NCED1/2* expression level and using ABA inhibitor cause early bud release in potato (Destefano-Beltrán et al., 2006). Yang et al. (2019) identified 17 GAST genes which are the potential candidate genes involved in bud dormancy. *PpyGAST1* gene regulates the ABA signal transduction pathway, and its expression is correlated with bud break in pear.

## Abscisic Acid Mediates the Synthesis of Ethylene and Crosstalks With Other Hormones

Evidence from several research studies confirms the complexity of the fruit ripening process, which is regulated by a complex network of interactions between different phytohormones. Data from genomic studies and high-throughput sequencing have helped to understand much better the expression of genes associated with hormones (Kumar et al., 2013). Phytohormone homeostasis is essential for fruit development and ripening, where the orchestrated crosstalk between different phytohormones regulates specific developmental signals for fruits to become edible.

### Interdependence of Abscisic Acid and Ethylene in Fruit Development

Complex interactions during fruit development decide the overall fruit quality whereby ethylene is documented to play a central role in the ripening process along with other phytohormones that interact and influence fruit physiology. As both ABA and ethylene are essential to plant hormones involved in fruit ripening, the interest in understanding the interaction between their signaling mechanisms is growing (Binder, 2020; Chen et al., 2020b). The plant hormones work *via* a complex network, and any change in one can alter the level of others in the system, which is why their concentrations keep changing accordingly. Many studies have reported the antagonistic effect of ABA and ethylene, but now we know that these two plant hormones can also act in a coordinated parallel manner stimulating each other’s biosynthesis ABA activates Calcium-dependent Protein Kinase 4 (CPK4) and CPK11 to stabilize 1-Aminocyclopropane-1-Carboxylate Synthase 6 (ACS6), by phosphorylating its C-terminus, and thus promoting ethylene biosynthesis (Luo et al., 2014; Li et al., 2019).

The role of ABA as an upstream regulator for modulating ethylene biosynthesis influencing fruit ripening was confirmed using tomato as the model system (Mou et al., 2016). Exogenous application of ABA was shown to regulate *LeACS2*, *LeACS4*, *LeACO1*, *LeGR*, and *LeETR6* genes of the ethylene synthesis pathway, suggesting a positive impact of ABA on ethylene production. It has also been hypothesized that ethylene may also be critical for the induction of ABA synthesis. The study of maturation-related transcription factors such as TAG1, NOR supported the TF-mediated interaction between *ABA and ethylene*. The study extends insight into the complex mechanism of ABA and ethylene interaction at the transcriptional level and the overall mechanism of tomato fruit ripening (Mou et al., 2016).

Genetic evidence is available illustrating the central role of NCED (9-*cis*-epoxycarotenoid dioxygenase) in ABA biosynthesis in plants (Iuchi et al., 2001). The NCED gene was cloned to study its expression in tomatoes to get insight into the mechanism of ABA and ethylene in fruit ripening (Zhang et al., 2009a). Cloning of cDNA sequences *LeNCED1* and *LeNCED2* encoding for NCED gene (from ABA pathway), as well as *LeACS2*, *LeACS4*, and *LeACO1* coding for ACC synthase and ACC oxidase (involved in ethylene biosynthesis), was done to evaluate the role of ABA in ethylene production. *LeNCED1* was thought to act as the first inducer responsible for initiating ABA biosynthesis at the onset of fruit ripening. External ABA treatment induced both the ACS and ACO gene expression, which eventually promoted ethylene synthesis and fruit ripening. The results concluded a clear relation between ABA and ethylene, suggesting the induction of ethylene biosynthesis by ABA through regulation of ACS and ACO genes in tomatoes. Also, ethylene was shown to have a role during later stages of ripening. The investigation reveals that the applications of ABA to mature fruit induce ethylene synthesis accelerated fruit coloring, and softening.

It is presumed that ethylene has little role to play in the ripening of non-climacteric fruits, but it was proved otherwise by a study done on grapefruit ripening (Sun et al., 2010). Grape berry ripening is majorly dependent on ABA, but interactive synergism and reciprocity between ABA and ethylene have been observed at the onset of grape berry ripening. ABA was found to regulate ethylene biosynthesis genes *via* signal transduction. ABA stimulated ethylene production in the véraison (mature berries). On harvesting, ABA levels increased with water loss from the pedicle, and ethylene production happened subsequently. Still, when water was supplied externally to the pedicle, ABA content in berries declined, and even ethylene production ceased to demonstrate the correlation between the two phytohormones. Similar results were observed in the case of other non-climacteric fruit such as strawberry and pepper. They observed ethylene mediated ABA accumulation in strawberry and activation of acyltransferase gene transcription due to overexpression of ethylene regular *FveERF* (FvH4_5g04470.1) (Tosetti et al., 2020). In pepper (*Capsicum*), ethylene was shown to regulate fruit color, influencing carotenoid biosynthesis positively. At the same time, ABA had a role in chlorophyll degradation, and hence de-greening of fruits (Hou et al., 2018a).

To know what modulates the interaction between ABA and ethylene, gene expression analysis with ABA deficient mutants and RNAi SlZFP2 lines were used in tomatoes. It was found that TF- SlZFP2 does the cross-talk between these two hormones. It regulates ABA biosynthesis by directly suppressing pathway controls fruit ripening by transcriptional suppression of the CNR, ripening regulator. ABA upregulated ethylene biosynthesis pathway genes (*LeACS1A*, *LeACS1*, and *LeACO1*) during early fruit growth (Weng et al., 2015). Henceforth, if ABA has an indispensable role in fruit ripening regulation, it probably lies because of its positive influence on basal ethylene production.

## Crosstalk Between Phytohormones Influencing Fruit Development in Plants

Phytohormones have thoroughly been investigated because of their functions in regulating several aspects of plant developmental stages (Bansal and Wani, 2021; Pandita and Wani, 2021). There is enough evidence to suggest that role of these hormones has no limitations to a specific stage, and an interlinked network of these hormones is associated with the regulation of various aspects of fruit development (Fenn and Giovannoni, 2021; Xiong et al., 2021). They synchronize signals between seed and surrounding tissue, regulating the whole process of fruit development starting from fruit setting, growth, maturation, and ripening (Fuentes et al., 2019; Saddhe et al., 2021). The advance in genomics has assisted in understanding the overlapping patterns of gene expressions involved with these hormonal actions across different crop plant species (Zhang et al., 2009b; Gao et al., 2018).

The initial fruit setting is controlled by a coordinated action of auxin, cytokinin, and GA which regulate cell division (Mariotti et al., 2011). It has been observed that combined usage of these phytohormones can promote fruit growth even when there is a lack of fertilization, pointing at the importance of hormonal level interplay in plants for fruit development (Vivian-Smith and Koltunow, 1999). Most studies have focused on the action of GAs and auxins in fruit settings as in the case of tomatoes, it was seen that auxins act by inducing GA biosynthesis (Serrani et al., 2008; Ruan et al., 2012). Auxin response factor *SlARF7* is considered as the critical player in auxin-GA crosstalk and controls their signaling pathway (de Jong et al., 2011; Carrera et al., 2012). According to Fu et al. (2008) brassinosteroids possibly also have a crucial role to play in fruit sets though no detailed study is available. Therefore, the fruit setting relies on a delicate balance between plant hormones, through which the hormones play a critical role depending on the plant species.

During fruit growth and maturation, cell enlargement, and accumulation of storage products take place. Through transcriptomics study, we now know that multiple cell wall proteins, sugar transporters, and solubilizing enzymes get upregulated during maturation. The responsible genes for these enzymes are in turn regulated by phytohormones- auxins, GAs, and ABA (Carrera et al., 2012; Nitsch et al., 2012). Auxin and cytokinin appear to be the main regulators of fruit maturation. Genetic studies have shown that the tomato *ripening inhibitor* (*rin*) mutant has increased auxin and cytokinin levels compared to the wild-type fruit (Davey and Van Staden, 1978; Rolle and Chism, 1989).

Fruit maturation is a transitional phase that arises when auxin and GA levels subside along with a simultaneous rise in ABA and ethylene content. Progression of fruit ripening is accompanied by a cell wall and color change causing fruit softening (Klee and Giovannoni, 2011) and majorly takes place through ABA and ethylene as illustrated in Figure 3 (Setha, 2012). Climacteric fruits have a proven role in ethylene, but auxins such as IAA have been reported to do the crosstalk with ethylene during the onset of ripening in tomato and peach. Also, ABA amount is seen increasing before ethylene which is supported on the basis that the former performs a role in the ethylene biosynthetic pathway too (Zhang et al., 2009b). Different traits of fruit ripening, such as color change or sugar accumulation, respond differently to various hormones, and no one rule applies to all. Color change during fruit ripening takes place due to the loss of chlorophyll and the formation of carotenoids and anthocyanins. ABA and/or ethylene again have a role in the color change of fruits whereby the production of carotenoids is found ethylene-regulated in tomatoes. On the other hand, Brassinosteroids are seen to inhibit color change in grape and strawberry (Symons et al., 2006; Chai et al., 2013).

What was once considered a simple antagonism of hormones like ABA vs. GA or ethylene in fruit development is now more aptly referred to as systematic crosstalk between various phytohormones. Some areas of fruit development have been extensively worked upon, but many gaps still exist in our complete understanding. However, one thing is clear that instead of individual role play of hormones, there is a well-controlled and coordinated interplay of phytohormones responsible for fruit development in plants.

## Conclusion

The burgeoning population and the effect of climate change have compelled researchers to be critical about food and nutritional security. Therefore, studying the physiology of the crop plants in context to the role of phytohormones will help elucidate their exact position and develop high-yielding, nutritious and climate-resilient crop varieties. Along with their significant role in stress, tolerance phytohormones are the central player of several critical physiological functions in plants. Similarly, the role of ABA has been proved against various stresses mainly, abiotic stresses and in different physiological processes in plants. ABA shares a crucial part of its multiple functions in crop plants during fruit developmental and ripening phases. These roles have elaborately been explained, along with reviews of several different studies in this context. ABA and other phytohormone have been instrumental in this process, and further studies are essential to elucidate this crosstalk. Furthermore, the amalgamation of a novel interdisciplinary approach is vital to dissect the coordination of ABA with other hormones in the regulation process of fruit development and ripening.

## Author Contributions

All authors listed have made a substantial, direct, and intellectual contribution to the work and approved it for publication.

## Conflict of Interest

The authors declare that the research was conducted in the absence of any commercial or financial relationships that could be construed as a potential conflict of interest.

## Publisher’s Note

All claims expressed in this article are solely those of the authors and do not necessarily represent those of their affiliated organizations, or those of the publisher, the editors and the reviewers. Any product that may be evaluated in this article, or claim that may be made by its manufacturer, is not guaranteed or endorsed by the publisher.
